# From Struggling (With) Screening Tests to Mouse Models of Depression‐Relevant Neurobehavioral States

**DOI:** 10.1002/cpz1.70312

**Published:** 2026-01-30

**Authors:** Christopher R. Pryce

**Affiliations:** ^1^ Adult Psychiatry and Psychotherapy University Hospital of Psychiatry and University of Zurich Zurich Switzerland

**Keywords:** chronic social stress, forced swim test, generalized threat, RDoC, tail suspension test

## Abstract

A major, serendipitous psychiatric discovery is monoamine‐transporter reuptake inhibition as an antidepressant mechanism of action. Chronic treatment with such antidepressants is efficacious, with onset requiring 1‐2 weeks, in many but by no means all patients with major depressive or another stress‐related neuropsychiatric disorder. The forced swim test (FST) in rats and mice involves the acute, moderate stressor of placement in a container of water: at test onset, the predominant reaction is swimming, interpreted cautiously as active “struggling”; over minutes, this is replaced by floating, described objectively as immobility. Acute administration of monoamine transporter inhibitors immediately prolongs “struggling.” Although this readout is of behavioral pharmacological interest, the FST has no (back‐)translational relevance to the neurobiological and neuropsychological symptoms/states of stress‐related psychiatric disorders. The persistent adoption of the FST to measure “depression‐like state,” based on interpretation of immobility as “despair,” “helplessness,” or “passive coping,” is a major weakness in applied behavioral neuroscience. Rodents do have a concept of learned uncontrollability, such that tests showing this depict an adaptive, not a “depression‐like,” state. Recent psychiatry‐neuroscience initiatives, such as the Research Domain Criteria framework, increase the accessibility of specific, transdiagnostic symptoms/states to behavioral neuroscience methods, thereby facilitating the establishment of animal models. Such animal models must incorporate clinically valid (1) etiological factors, such as prolonged psychosocial stress, and (2) neurobehavioral readouts with face and construct validity for specific symptoms/states. Increased reactivity to an acute threat, measured as increased Pavlovian aversion learning‐memory (PALM), is a neurobehavioral state common in major depression and other disorders. Mice that have undergone chronic social stress exhibit generalized excessive PALM. Therefore, although stating that the FST measures “depression‐like state” is erroneous, mouse models of specific symptoms/states can be achieved by back‐translating etiology and neurobehavioral readouts. Though complex and moderately severe, such models have the potential to provide much‐needed benefits in terms of preclinical neuropharmacological target discovery and validation. © 2026 The Author(s). *Current Protocols* published by Wiley Periodicals LLC.

## INTRODUCTION

Neuropsychiatric disorders such as major depressive disorder (MDD), post‐traumatic stress disorder (PTSD), and schizophrenia (SCZ) are complex and heterogeneous (APA, 2013). Immense research effort is required to meet the challenges of understanding their etio‐pathophysiology and discovering efficacious therapies for their major, often transdiagnostic, symptoms. One indispensable part of this effort is applied behavioral neuroscience research with animal models, in which ethically justifiable experiments are conducted to elucidate the neurobiological bases of symptom‐relevant neurobehavioral states and to discover and validate molecular targets for novel pharmacotherapies. Ethical justification means that the benefits in medical knowledge outweigh the costs in terms of stress and pain experienced by the animals; this will only be the case when the experiments are conducted using animal models with validity. In the current context, validity refers to the relevance of the animal model to the human neuropsychiatric disorder symptom with respect to the application of major environmental and/or (epi)genetic risk factors (etiological validity); measurement of psychological/behavioral dysfunction (face validity); and measurement of altered neurobiological state (construct validity). In the case of neuropsychiatric disorders, applied behavioral neuroscience has had profound difficulty in adhering to the principles of animal model validity, and this has been the case for many years.

The aims of this overview are to present: (1) The rodent behavioral paradigms, forced swim test and tail suspension test, that are acute screening tests for the antidepressant mechanism of action (MoA) monoamine‐transporter reuptake inhibition, but have been misinterpreted and misapplied as rodent models of a “depression‐like state” of “helplessness” or similar. (2) A rodent behavioral paradigm which demonstrates that rodents can indeed learn when a specific aversive environment is controllable or uncontrollable, but also that the latter behavioral state is adaptive and not “depression‐like.” (3) Ongoing initiatives to increase the accessibility, or back‐translatability, of specific neuropsychiatric disorder symptoms/states/constructs to animal models and applied behavioral neuroscience. (4) A back‐translated mouse model in which chronic psychosocial stress leads to a neurobehavioral state of generalized excessive aversion reactivity, relevant to MDD and other stress‐related psychiatric disorders.

## RODENT ANTIDEPRESSANT SCREENING TESTS ARE NOT MODELS OF DEPRESSION‐LIKE STATE

The forced swim test (FST) in rats was first described in 1977 (Porsolt et al., 1977) and comprised a 15‐min placement in a cylinder of water followed 24 hr later by a 5‐min replacement in the water cylinder. Drugs from the two antidepressant classes established at that time, monoamine oxidase inhibitors and tricyclic monoamine‐transporter reuptake inhibitors, were given three times during this intervening 24‐hr period and were seen to dose‐dependently reduce the duration of immobility in the 5‐min test. In the case of the FST in mice, there is only one placement in water, typically for 6 min, during which swimming, or climbing attempts, predominate at the beginning and floating (immobility) predominates at the end, with compounds being administered acutely prior to the test (Cryan et al., 2002; Porsolt, 2000). The tail suspension test (TST) in mice, first described in 1985, comprises suspending the mouse by taping the tip of its tail to a lever for 6 min; after an initial period of running movements, torsion, and jerks, immobile hanging predominates (Steru et al., 1985). For the rat FST, Porsolt et al. write: “The method is based on the observation that a rat, when forced to swim in a situation from which there is no escape, will, after an initial period of vigorous activity, eventually cease to move altogether making only those movements necessary to keep its head above water. We think that this characteristic and readily identifiable behavioral immobility indicates a state of despair in which the rat has learned that escape is impossible and resigns itself to the experimental conditions. This hypothesis receives support from results…. which indicate that immobility is reduced by different treatments known to be therapeutic in depression….” (Porsolt et al., 1977). Therefore, rodent FST as a monoamine‐based antidepressant screening test and the hypothesis of rodent FST as a model of “depression‐like state” were convoluted in the original description of the FST, in *Nature*, and have remain convoluted since. Porsolt et al. thought/hypothesized that immobility indicated despair, an opinion that has prevailed until today, and the more it has been stated, the more it has become regarded as fact. This anthropocentric approach to applied behavioral neuroscience is in stark contrast to the objective approach of rigorous hypothesis testing and parsimonious interpretation applied in rodent behavioral tests in experimental psychology. Examples of the latter include the establishment of tone‐foot shock pairing leading to freezing behavior as a paradigm with which to study Pavlovian aversive association learning‐memory (Rescorla, 1988) and of the five‐choice serial reaction time task as a paradigm with which to study sustained visuospatial attention (Robbins, 2002). In this regard, it is important to note that Steru et al. were objective in their interpretation of the TST: “a normal animal submitted to an insoluble, aversive situation alternates between two kinds of behaviors, agitation and immobility. These can be named *searching‐behavior* characterized by intense motor activity and expense of energy, and *waiting‐behavior* with immobility and energy saving. The choice sequences between these kinds of behaviors can be named as the *searching‐waiting strategy*. The [TST] data support the assumption that antidepressant drugs modify the balance between these forms of behavior in the favor of searching” (Steru et al., 1985). That is, the original report of the TST did not succumb to the “depression‐like state” fallacy, but numerous publications since have used this mistaken reasoning to justify their use of the TST for purposes other than monoamine‐based antidepressant screening.

## RODENT UNCONTROLLABILITY

When Porsolt et al. (1977) wrote that “behavioral immobility indicates a state of despair in which the rat has learned that escape is impossible and resigns itself to the experimental conditions,” they were clearly being anthropocentric, as have been the hundreds, at least, of authors of subsequent papers who have claimed to deploy the FST as a model of a “depression‐like state.” Leaving aside the complex emotional state of despair, it is certainly reasonable to propose that a rat or mouse can learn that a certain behavior is without consequence: specifically, in the case of the FST, that swimming in water does not lead to exiting water. Now, imagine that the FST has been modified and that at various time points of the test, a substrate, such as a rope or platform, is provided that the rat or mouse can use to climb and exit the water. If the likelihood that the animal will use the substrate decreases the longer it has been floating in the water, then we could propose that it is in a state in which it has learned that any behavior will be without consequence with respect to exiting the water. This would be a state of specific learned aversive uncontrollability (Maier & Seligman, 1976). Although this is not a “depression‐like state”—the learned uncontrollability is accurate and adaptive up to that time point—it is at least a demonstrated neurobehavioral state based on aversive experience. Although the FST lacks this aspect of assessing (un)controllability, it is assessed in another rodent paradigm, typically referred to as the learned helplessness (LH) paradigm, and more parsimoniously as the specific learned aversion uncontrollability (SLAU) paradigm.

**Figure 1:**
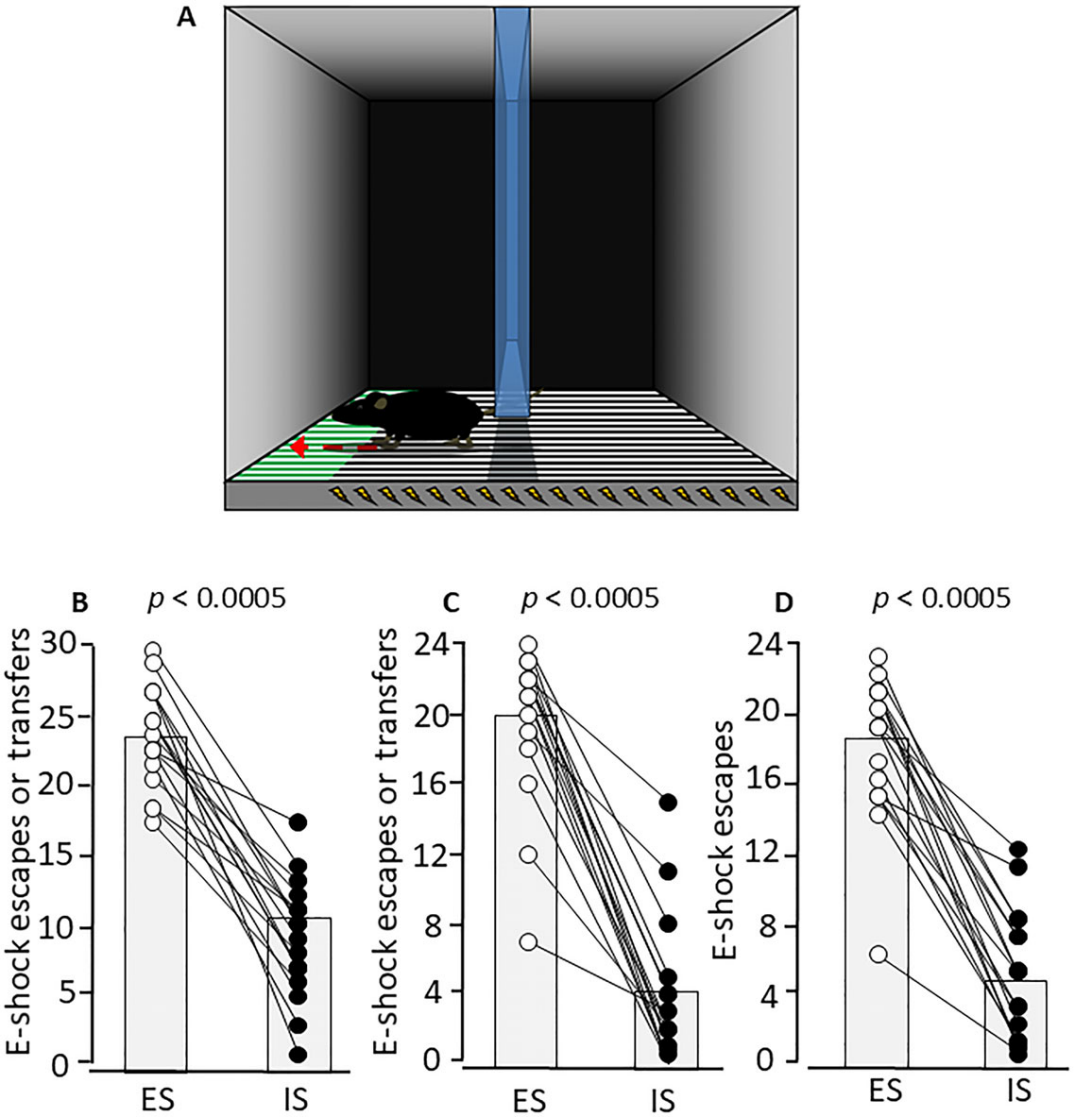
**A paradigm for specific learned aversion uncontrollability in mice**. (**A**) Mice are placed in an arena with a floor of electrifiable stainless‐steel rods, and with a central divider running from back to front and raised off the ground, allowing transfer between the left and right compartments. In the sketch, the mouse is in the right compartment at the onset of e‐shock: during pre‐exposure sessions, in the case of an escapable e‐shock (ES) mouse, transfer under the central divider and crossing with the head beyond the first 70% of the left compartment constitutes an escape response and results in termination of the e‐shock; in the case of the inescapable e‐shock (IS) littermate, the same behavior is without consequence and the duration of the e‐shock is the escape latency of the ES mouse. During the test session, in the case of both ES and IS mice, transfer under the central divider and crossing with the head beyond the first 70% of the left compartment (as shown) results in termination of the e‐shock. (**B**‐**D**) In littermate pairs of males (*n* = 17), one littermate was assigned to ES and the other to IS. They underwent three daily pre‐exposure sessions, in which ES mice received escapable e‐shocks of 0.15 mA × maximum 4‐5 sec, and the escape latencies were used as the duration of inescapable e‐shocks administered to the IS mice. This was followed by an escape test in which both ES and IS mice received escapable e‐shocks of 0.15 mA × maximum 4 sec. (**B**) Pre‐exposure session 2: in 30 trials of 0.15 mA × 5 sec maximum, number of escapes by ES mice and number of transfers (“escape attempts”) by IS mice. (**C**) Pre‐exposure session 3: in 24 trials of 0.15 mA × 4 sec maximum, number of escapes by ES mice and number of transfers by IS mice. (**D**) Escape test: in 24 trials of 0.15 mA × 4 sec maximum, number of transfers by ES and IS mice. *p* values are for paired *t*‐tests. For further details, see Pryce et al. (2012).

The main principle underlying the LH/SLAU paradigms is that rodents are, in a first, pre‐exposure phase, assigned to two groups that receive identical aversive stimulation of the same duration except that for one group the stimuli are escapable (escapable e‐shocks, ES) and for the other group they are inescapable (inescapable e‐shocks, IS). In a second, test phase, both the ES and IS groups are exposed to escapable aversive stimuli, and the LH/SLAU effect is evident in the reduced or slower escape responses by the IS group. The LH paradigm was first described, for rats, in the early 1970s (Maier & Seligman, 1976). In the first phase, tail electroshock was used as the aversive stimulus and wheel turning as the operant behavior that terminated the tail e‐shocks in the ES group; the escape latency for each ES subject provided the e‐shock duration for the paired/yoked IS subject, so that ES/IS e‐shock durations were equal. For the second phase, a two‐way shuttle box was used, and foot e‐shock was now the aversive stimulus, with compartmental transfer via a barrier or doorway the operant escape response: IS rats had a longer average escape latency than did ES rats. Similar experiments were conducted in mice starting in the late 1970s (Anisman et al., 1978, 1979; Anisman & Merali, 2001): many aspects of the paradigm were the same as those applied in rats, and the main difference was that aversive stimulus and operant escape response were the same at each phase—typically, foot e‐shock and compartmental transfer in a two‐way shuttle box. In these original versions of both the rat and mouse paradigms, in both phases the maximum duration of each e‐shock was long, up to 30 sec. In 2012, my laboratory published a mouse SLAU paradigm in which the maximum duration of each e‐shock in both phases was only 5 sec; this was sufficient to enable ES mice to escape most first‐phase trials. After three consecutive daily sessions of foot e‐shock in the two‐way shuttle box in the first phase, in the second, test phase, IS mice made substantially fewer foot e‐shock escape responses than did ES mice in the two‐way shuttle box (Fig. 1; Pryce et al., 2012).

Therefore, using paradigms with an appropriate control condition, it can be shown that rats and mice do have a concept of learned (un)controllability; as discussed elsewhere, this is a neurobehavioral state with emotional, motivational, and cognitive components (Pryce et al., 2011, 2012). When the aversive stimuli and the operant behavior required to escape are similar or identical in both phases of the paradigm, learned uncontrollability is an adaptive response and not a “depression‐like state” or depression‐relevant neurobehavioral state. Nonetheless, it is interesting to consider the data obtained when these paradigms have been applied for monoamine‐based antidepressant screening. In rats, it was reported for tricyclic antidepressants and selective serotonin reuptake inhibitors (SSRIs) that acute administration led to increased test phase escape behavior in the IS group specifically, but there are also reports of no effect from such acute administration (Pryce et al., 2011).

## INCREASING THE DIALOG BETWEEN NEUROPSYCHIATRIC DISORDERS AND APPLIED BEHAVIORAL NEUROSCIENCE

Although attempts to design a rodent model of “depression‐like state” were and will always be unrealistic, there will inevitably be pressure to do so for as long as the focus of psychiatric research is on the heterogenous, nosological entity of MDD. This is one of the major reasons why it is so important that relatively recent initiatives have redirected research focus away from nosological psychiatric entities and toward specific neurobehavioral functions and the pathologies thereof. Such an approach renders neuropsychiatric disorder symptoms and states amenable to study using the methods of molecular, cellular, systems, and behavioral neuroscience; this includes human studies, animal studies, and (back‐)translation between them. Pioneering in this regard have been the Research Domain Criteria (RDoC) framework of the U.S. National Institute of Mental Health (www.nimh.nih.gov) and also, more recently, the Precision Psychiatry Roadmap of the European College of Neuropsychopharmacology (Kas et al., 2025). In the case of the RDoC framework, six domains of psychological/neurobiological processes are identified, with those of most and equable relevance to MDD being negative valence systems and positive valence systems. Negative valence systems are responsible for the processing of aversive events/stimuli, which include acute actual threats, acute potential threats, and chronic exposure to acute and/or potential threats, and the responsiveness to and learning about such events/stimuli. With respect to MDD symptoms, these systems interface with the core symptom of depressed mood (APA, 2013). Furthermore, chronic exposure to acute and/or potential threats, and particularly those that are (perceived as) uncontrollable, is a major etiological risk factor for the onset and maintenance of MDD (Kendler et al., 1999; Pizzagalli, 2014; Sanacora et al., 2022). Positive valence systems are responsible for the processing of rewarding events/stimuli, and include reward anticipation/motivation, learning, valuation, and responsiveness with pleasure. These symptoms interface with the core MDD symptom of diminished interest or pleasure in daily activities (APA, 2013).

Given this fundamental shift in approach, it becomes clear that the term “depression‐like state” is redundant; depression/MDD comprises specific altered states with respect to aversion processing and reward processing (and, indeed, with respect to other common symptoms such as disturbed sleep). Accordingly, animal models of these states also need to be precise with respect to the altered emotional state that is being modeled. In the next section, I present a specific example of such a model of a depression‐relevant neurobehavioral state, as developed in mice.

## MOUSE MODELS OF DEPRESSION‐RELEVANT NEUROBEHAVIORAL STATES

### Chronic Social Stress as Environmental Etiology

**Figure 2:**
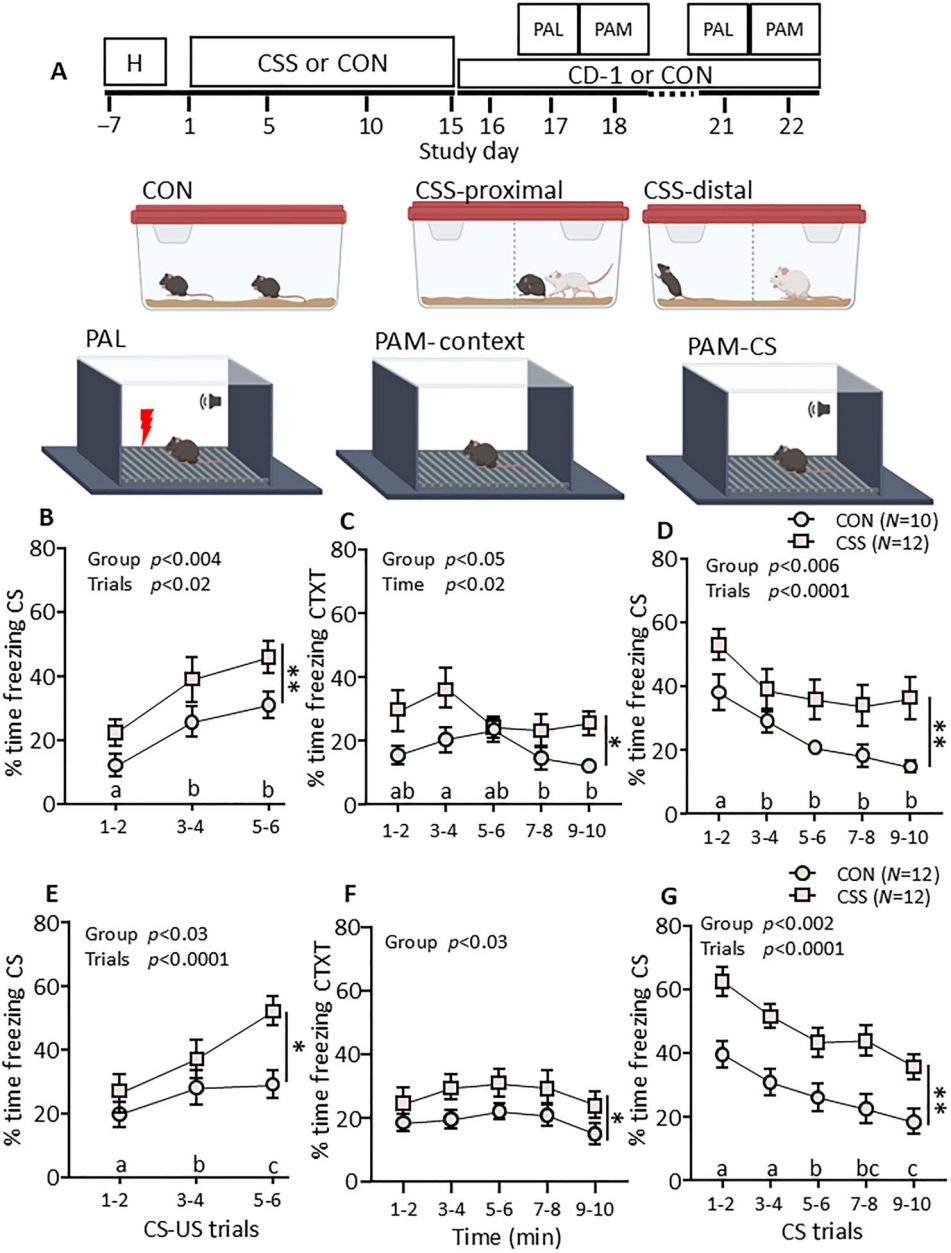
**Chronic social stress‐induced excessive PALM**. (**A**) Experimental design for the CSS‐PALM model. H, handling; CSS or CON, chronic social stress or control handling; CD‐1 or CON, CSS mice maintained in distal contact with a CD‐1 mouse or control handling; PAL, Pavlovian aversion learning test; PAM, Pavlovian aversion memory tests for context and auditory CS. Top, timeline: Mice underwent CSS/CON on days 1‐15, CS‐US conditioning (PAL) on day 17 (Expt. 1, **B**) or day 21 (Expt. 2, **E**), and PAM testing on day 18 (Expt. 1, **C**, **D**) or day 22 (Expt. 2, **F**, **G**). Middle, setup for chronic social stress (CSS): the BL/6J mouse and CD‐1 mouse are placed together (CSS‐proximal) in the same compartment of the CD‐1 mouse home cage, for a maximum of 10 min or 60 sec attack or 10 sec biting, whichever occurs soonest, and are then separated by the divider for 24 hr (CSS‐distal). The next day, each BL/6J (CSS) mouse is rotated to the next CD‐1 mouse cage and then exposed to proximal and distal stressors. This continues for a total of 15 days, such that each CSS mouse encounters 12‐15 different CD‐1 mice and cages. CSS mice remain distally exposed to the CD‐1 mouse they are last paired with until the end of the experiment, without any further proximal stress/attacks. Control (CON) BL/6J mice are maintained as pairs of male littermates and are handled daily as a control for the handling required with CSS mice. Bottom, setup for PALM testing: Between 1 and 6 days after the 15‐day CSS/CON protocol, mice undergo a Pavlovian aversion learning (PAL) test comprising placement in an arena (context) and exposure to six pairings of a tone conditioned stimulus (CS, 6.5 kHz × 85 dB × 20 s) and an e‐shock unconditioned stimulus (US, 0.15 mA × 2 s), with 120‐sec inter‐trial intervals. Between 1 and 10 days after the PAL test, mice undergo a Pavlovian aversion memory test comprising placement in the same context and a PAM‐context test in the absence of the CS followed by a PAM‐CS‐context test in which the CS is presented for 10 × 30 sec with 90‐sec inter‐trial intervals. (**B**‐**D**) CSS‐PALM experiment 1, with 1 day between CSS and PAL test and 1 day between PAL test and PAM test. (**B**) At PAL testing, acquisition of freezing during CS trials (measured as % of time spent freezing during each CS) was higher in CSS mice than in CON mice; % time freezing during CS was higher in trials 3‐4 and 5‐6 than trials 1‐2. Inset: during the US, there was no Group effect on reactivity distance, suggesting that the pain experienced was similar in CSS and CON mice. The US reactivity distance decreased across trials. (**C**) In the context memory test, % time freezing was higher in CSS mice than in CON mice. (**D**) In the CS PAM test, % time freezing during CS trials was higher in CSS mice than in CON mice. % time freezing decreased after CS trials 1‐2. (**E**‐**G**) CSS‐PALM experiment 2, with 6 days between CSS and PAL test and 1 day between PAL test and PAM test. (**E**) At PAL testing, acquisition of % time spent freezing during CS trials was higher in CSS mice than CON mice; % time freezing during CS increased progressively across trials. Inset: during the US, there was no Group effect on reactivity distance, suggesting that the pain experienced was similar in CSS and CON mice. The US reactivity distance decreased across trials. (**F**) In the context memory test, % time freezing was higher in CSS mice than in CON mice. (**G**) In the CS PAM test, % time freezing during CS trials was higher in CSS mice than in CON mice. % time freezing decreased progressively across CS trials. For repeated measures of trials and time bins, pairs of trials or bins denoted by different letters were significantly different from each other at *p* < 0.05 at least: e.g., a vs. b = significantly different, a vs. ab = not significantly different. For further details, see Poggi et al. (2025).

For an animal model of a depression‐relevant neurobehavioral state to be valid requires that the experimental group of subjects undergoes environmental events resembling those undergone by human patients presenting with the state/symptom of interest. Psychosocial stressors are the most relevant events in this regard, and their chronicity, uncontrollability, and unpredictability are key characteristics that need to be recapitulated in the model. Chronic social stress (CSS; Fig. 2A) involves placing young‐adult male C57BL/6J mice in the home cages of ex‐breeder aggressive‐dominant male CD‐1 mice, which contain a central divider that is perforated and allows olfactory, auditory, and visual, but not tactile, interaction between a CSS mouse and a CD‐1 mouse placed on either side of it. On day 1, the two mice are placed on the same side of the divider and the CSS mouse is attacked by the CD‐1 mouse; the mice are kept together for a cumulative total of 60 sec of attack or 10 min maximum, whichever occurs sooner, and this is followed by separation and continuous distal interaction/stimulation via the central divider. Over 15 days, the CSS mice are rotated between the CD‐1 mice, so that each CSS mouse is placed in the home cage of, and attacked by and distally exposed to, a different CD‐1 mouse across each 24‐hr period. Therefore, the chronicity of CSS is 15 days. When the CSS mouse is attacked by a CD‐1 mouse, it displays submissive behavior (body posture, vocalization), but this does not result in termination of attack by CD‐1 mice, so that CSS mice are repeatedly exposed to an uncontrollable stressor and then to the distal stimuli associated with that stressor. The CD‐1 mice differ in their behavior, with some attacking immediately and others delaying attack and inspecting and even grooming the CSS mouse prior to attack onset, so that the stressor is unpredictable over days. Control mice are matched in age and body weight with the CSS mice and are caged together in littermate pairs (Fig. 2A). Although separation by a central divider would control for that aspect of the CSS procedure, this would constitute a social stressor for littermates. Accordingly, the littermate pairs can be considered as a comparison group, maintained under conditions typical for mice in the colony, rather than a control group in a strict sense. The procedural details of CSS are explained in (Sigrist et al., 2024).

Although the focus of this overview article is to describe mouse models for behavioral states relevant to MDD symptoms, clearly it is essential that the environmental event, in this case CSS, also leads to a symptom/state‐relevant shift in the basal state of the brain circuitry underlying the translational behavioral state. To date, some of the major neurobiological effects of CSS that we have demonstrated are increased resting‐state functional connectivity between amygdala‐prefrontal cortex, amygdala‐cingulate cortex, and amygdala‐ventral hippocampus (Grandjean et al., 2016); increased serotonin (5‐HT) receptor _1A_ level in dorsal raphe nucleus and decreased 5‐HT_2A_ level in amygdala and ventral tegmental area (Carneiro‐Nascimento et al., 2021); and reduced number of amygdala glutamate neurons expressing the immediate‐early‐gene protein c‐Fos after aversion learning and aversion memory recall (Poggi et al., 2025).

### Generalized Excessive Pavlovian Aversion Learning‐Memory

In the case of MDD, and also PTSD and generalized anxiety disorder, relative to healthy control subjects, excessive Pavlovian aversion learning‐memory (PALM) about succinct threat/aversion, associated with increased amygdala and hippocampal blood oxygenation level‐dependent (BOLD) imaging activity, constitutes a transdiagnostic neurobehavioral state marker of relevance to symptoms such as low mood, rumination, and increased reactivity associated with a traumatic event. Typically, electroshock or heat to the forearm constitutes the unconditioned stimulus, a visual stimulus on a screen or a tone the conditioned stimulus, and skin conductance the conditioned response (Beckers et al., 2023; Lissek et al., 2005, 2014; Nissen et al., 2010; Suarez‐Jimenez et al., 2020). In the case of CSS mice, PALM is also excessive relative to that in control mice, such that CSS‐PALM constitutes a robust and reproducible back‐translational model for hyper‐reactive negative valence processing (Fig. 2). In Pavlovian aversion learning (PAL; Fig. 2A), a mild electroshock to the paws is the unconditioned stimulus (US, foot shock) and a tone is the conditioned stimulus (CS), with six CS‐US pairings being presented to the mice in an arena (context; Fig. 2A). The conditioned response is freezing—the absence of any detectable movement—which is an adaptive response in the context of predator avoidance. The CSS mice acquire more freezing than do control mice—i.e., they spend a higher percentage of the time that the CS is presented in a state of freezing—indicating that the emotional salience of the US, and therefore of the CS, is increased by CSS: that is, psychosocial stress leads to a generalized increase in aversion salience (Fig. 2B and E). This is the case when PAL is conducted once between 1 day and 5‐6 days after completion of the 15‐day CSS protocol, with it being imperative that the CSS mice are kept in the cages with the CD‐1 mice, separated by the divider and without further attack sessions, across the behavioral testing period. For Pavlovian aversion memory (PAM), mice are returned to the same context, where, first, contextual freezing is measured: the CSS mice express more freezing than do control mice (Fig. 2C and F). Mice are then presented with CS trials and again freezing is the conditioned response of interest: the CSS mice express more freezing than do control mice, i.e., they spend a higher percentage of the time that the CS is presented in a state of freezing, indicating that the consolidation and recall of the CS is increased by CSS (Fig. 2D and G). The procedural details of the CSS‐PALM model are explained in Sigrist et al. (2024).

**Figure 3:**
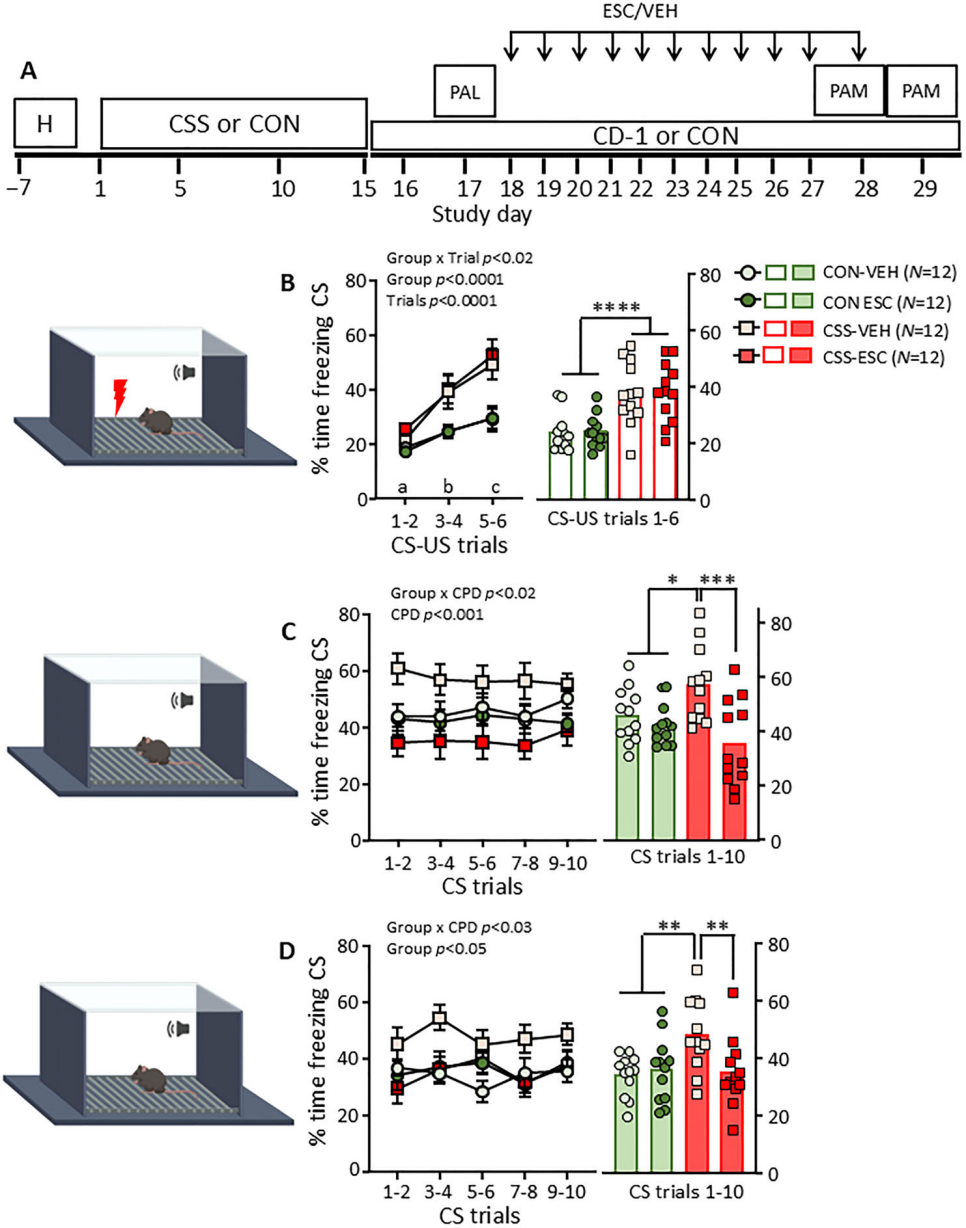
Reversal of chronic social stress‐induced excessive PALM by sub‐chronic escitalopram. (**A**) Experimental design for the application of the CSS‐PALM model to investigate the preclinical efficacy of the selective serotonin reuptake inhibitor antidepressant escitalopram (ESC). H, handling; CSS or CON, chronic social stress or control handling; CD‐1 or CON, CSS mice maintained in distal contact with a CD‐1 mouse or control handling; PAL, Pavlovian aversion learning test; PAM, Pavlovian aversion memory test. Mice underwent CSS/CON on days 1‐15, CS‐US conditioning on day 17, per os administration of escitalopram (ESC, 3 mg/kg) or vehicle (VEH) on days 18‐28, and PAM testing on days 28‐29. Left‐hand graphs present group‐dose mean ± SEM data for pairs of consecutive trials, and right‐hand graphs present per mouse mean values for all pairs of trials and the overall mean. (**B**) At PAL testing, % time freezing during CS was higher in CSS than in CON mice; percent time freezing during CS increased progressively across trials (CS‐US trial main effect: *F*_2,88_ = 27.45, *p* < 0.0001). CSS and CON mice were allocated to ESC and VEH by counterbalancing on mean % time spent freezing. (**C**) In the CS/context memory test on day 28, there was a Group × Dose interaction effect: % time freezing during CS trials was higher in CSS‐VEH mice than in CON mice and lower in CSS‐ESC mice than in CSS‐VEH mice. (**D**). In the CS/context memory test on day 29, there was a Group × Dose interaction effect: % time freezing during CS trials was higher in CSS‐VEH mice than in CON mice and lower in CSS‐ESC mice than in CSS‐VEH mice. For repeated measures of trials and time bins, pairs of trials or bins denoted by different letters were significantly different from each other at *p* < 0.05 at least: e.g., a vs. b = significantly different, a vs. ab = not significantly different. For further details, see Poggi et al. (2025).

The CSS‐PALM model has predictive validity for sub‐chronic administration of the SSRI escitalopram: that is, in CSS mice specifically, 10 days of escitalopram administration between PAL and PAM reduced the time spent freezing (Fig. 3). The model has also been applied to provide evidence for preclinical validation of transient receptor potential canonical channel 4 and 5 inhibition and somatostatin receptor 4 agonism (Poggi et al., 2025).

### Concluding Remarks

The first aim of this overview is to clarify that although the rodent FST and TST are behavioral assays in which acute administration of monoamine‐reuptake inhibition leads to increased activity, there is no foundation for interpreting this observation as evidence that inactivity in the tests indicates an underlying “depression‐like state”, and that interpretation does not constitute objective, evidence‐based translational neuroscience. Next, the rodent paradigm of specific learned aversive uncontrollability is presented. Whereas learning that a specific aversive stimulus cannot be escaped/avoided is an adaptive and not a depression‐relevant state, it can be demonstrated, using an appropriate control group, that rodents are able to process (un)controllability. This is highly relevant, given the etiological importance of (feelings of) stress uncontrollability to human MDD and other disorders. That there are currently major initiatives to build bridges between psychiatry and behavioral neuroscience is then highlighted: primarily, this involves focusing on specific symptoms and the relevant neurobehavioral states and stress‐related changes therein. The approach is commensurate with back‐ and forward‐translational studies in animal models, should these be available. As its final aim, this overview then presents a mouse model of a depression‐relevant behavioral state in the form of chronic social stress‐induced generalized and excessive aversion learning‐memory, which has predictive validity for sub‐chronic SSRI administration. Animal models with such etiological and face validities are integral to the applied translational neuroscience that will increase understanding of the etio‐pathophysiology of, and lead to discovery of novel pharmacotherapies for, major symptoms in MDD and other stress‐related neuropsychiatric disorders.

### Author Contributions

**Christopher Pryce**: Conceptualization; funding acquisition; resources; writing—original draft; writing—review and editing.

### Conflict of Interest

The author has no conflicts of interest. The research funder had no involvement in the design, conducting or interpretation of the research.

## Data Availability

The author has nothing to report.
